# Crystal structure of bis­[1,3-bis­(di­phenyl­phosphan­yl)propane-κ^2^ *P*,*P*′]platinum(II) dichloride chloro­form penta­solvate

**DOI:** 10.1107/S205698901500136X

**Published:** 2015-01-28

**Authors:** Bradley G. Anderson, Sarah A. Hoyte, John L. Spencer

**Affiliations:** a School of Chemical and Physical Sciences, Victoria University of Wellington, Wellington, New Zealand

**Keywords:** crystal structure, 1,3-bis­(di­phenyl­phosphan­yl)propane, platinum(II) complex

## Abstract

In the title compound, [Pt{Ph_2_P(CH_2_)_3_PPh_2_}_2_]Cl_2_·5CHCl_3_, the Pt^II^ cations, located on a centre of inversion, is coordinated by two chelating diphosphane ligands in a geometry which is close to square-planar. The chelate rings adopt a chair conformation. The Pt^II^ cations are arranged in layers separated by Cl^−^ anions as well as CHCl_3_ solvent mol­ecules. While this complex has been reported previously [Anderson *et al.* (1983 ▸). *Inorg. Chim. Acta*, **76**, L251–L252], this is the first time a structure has been determined.

## Related literature   

For structures of related group 10 *M* ^2+^ bis-diphosphane complexes, see: Pahor & Bruno (1977 ▸); Engelhardt *et al.* (1984 ▸); Ferguson *et al.* (1993 ▸); Berning *et al.* (1999 ▸); Raebiger *et al.* (2004 ▸); Fischer (2006 ▸). The corresponding Pt^0^ complex [Pt(dppp)_2_] [dppp is 1,3-bis­(di­phenyl­phosphan­yl)propane] has been reported by Asker *et al.* (1990 ▸). For a previous report of the title compound, see: Anderson *et al.* (1983 ▸). 
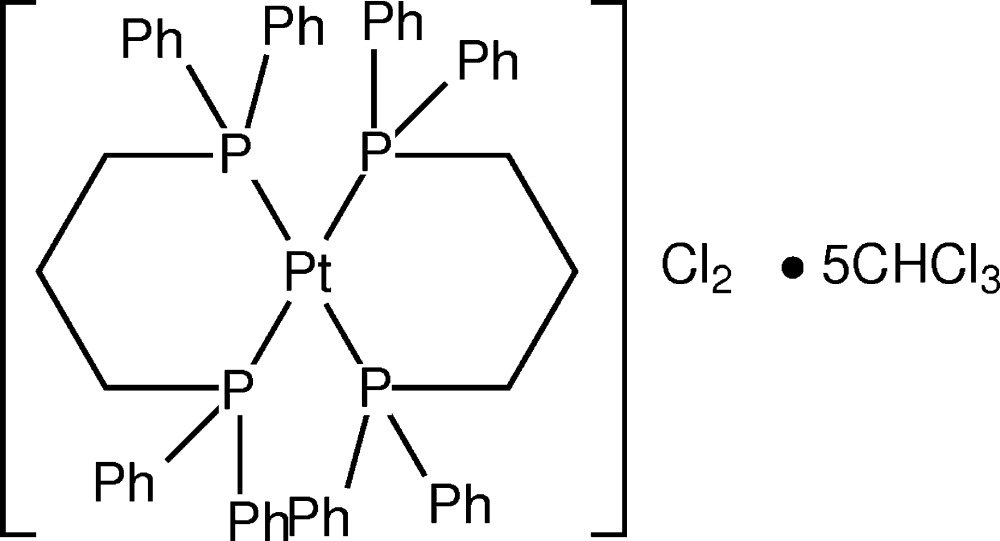


## Experimental   

### Crystal data  

[Pt(C_27_H_26_P_2_)_2_]Cl_2_·5CHCl_3_
*M* *_r_* = 1687.67
Orthorhombic, 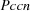

*a* = 26.2042 (9) Å
*b* = 15.3120 (5) Å
*c* = 16.7930 (5) Å
*V* = 6738.0 (4) Å^3^
*Z* = 4
Mo *K*α radiation
μ = 2.89 mm^−1^
*T* = 160 K
0.60 × 0.38 × 0.28 mm

### Data collection  

Bruker APEXII CCD diffractometer
Absorption correction: multi-scan (*SADABS*; Bruker, 2007 ▸) *T*_min_ = 0.562, *T*_max_ = 0.746
179974 measured reflections
10325 independent reflections
7580 reflections with *I* > 2σ(*I*)
*R*_int_ = 0.063

### Refinement  

*R*[*F* ^2^ > 2σ(*F* ^2^)] = 0.037
*wR*(*F* ^2^) = 0.073
*S* = 1.20
10325 reflections
385 parameters
H-atom parameters constrained
Δρ_max_ = 0.73 e Å^−3^
Δρ_min_ = −0.97 e Å^−3^

### 

Data collection: *APEX2* (Bruker, 2007 ▸); cell refinement: *SAINT* (Bruker, 2007 ▸); data reduction: *SAINT*; program(s) used to solve structure: *SHELXS97* (Sheldrick, 2008 ▸); program(s) used to refine structure: *SHELXL97* (Sheldrick, 2015 ▸); molecular graphics: *Mercury* (Macrae *et al.*, 2008 ▸); software used to prepare material for publication: *SHELXL97*.

## Supplementary Material

Crystal structure: contains datablock(s) I. DOI: 10.1107/S205698901500136X/gg2144sup1.cif

Structure factors: contains datablock(s) I. DOI: 10.1107/S205698901500136X/gg2144Isup2.hkl

**Fig. 1.**
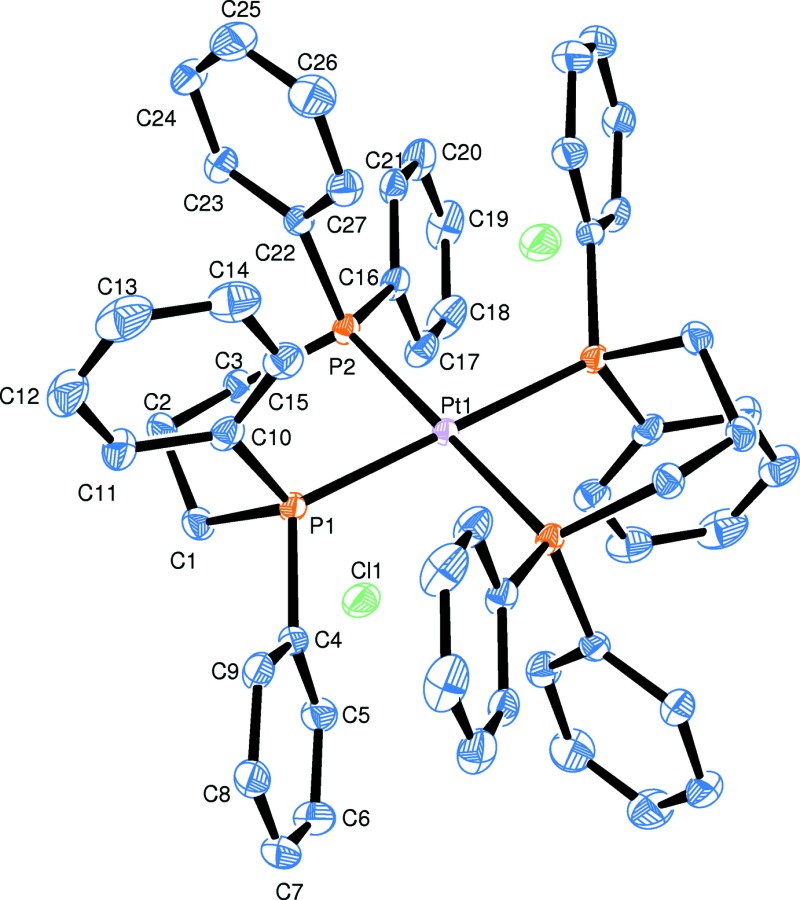
: ORTEP diagram of [Pt(Ph2P(C2H3)PPh2)2]Cl2 showing 50% probability ellipsoids. H atoms have been omitted for clarity.

ORTEP 2 2 3 2 2 2 . DOI: 10.1107/S205698901500136X/gg2144fig1.tif
*ORTEP* diagram of [Pt(Ph_2_P(C_2_H_3_)PPh_2_)_2_]Cl_2_ showing 50% probability ellipsoids. H atoms have been omitted for clarity.

CCDC reference: 1044833

Additional supporting information:  crystallographic information; 3D view; checkCIF report

## supplementary crystallographic information

### S1. Comment

When [PtCl_2_(SEt_2_)_2_] is reacted with bicyclopropylidene, this results in the formation of the β-chloroalkyl complex [Pt(C(CH_2_)_2_C(CH_2_)_2_Cl)Cl(SEt_2_)_2_] after 5 days. When dppp (Ph_2_P(CH_2_)_3_PPh_2_) was added in an attempt to make a phosphine β-chloroalkyl complex, the β-chloroalkyl ligand undergoes a β-chloride elimination to regenerate the alkene and [Pt(dppp)_2_]Cl_2_ is formed. While this complex has been reported previously (Anderson *et al.*, 1983), this is the first time a structure has been obtained (Fig. 1).

The asymmetric unit contains only half of the molecule, consisting of a complete dppp ligand as well as one of the Cl^-^ counter ions and half of the five CDCl_3_ solvent molecules. The platinum is close to square planar, with a P1—Pt—P2 angle of 87.23 (3)°. This is smaller than the corresponding angle in the Pt(0) complex [Pt(dppp)_2_] (97.76 (4)°) (Asker *et al.*, 1990). The Pt—P bond lengths are 2.3648 (7) and 2.3790 (8) Å, longer than those in [Pt(dppp)_2_] (2.286 (1) Å). The chelate ring has a 'chair' conformation, typical for dppp complexes. In [Pt(dppp)_2_]Cl_2_, the chelate two rings are rotated by 180° relative to each other, while in [Pt(dppp)_2_] the rings are rotated by 87.20 (2)° (according to the PtP1P2 planes). While [Pt(dppp)_2_]Cl_2_ crystallized as a CDCl_3_ solvate in the orthorhombic *Pccn* space group, [Pt(dppp)_2_] crystallized solvent-free from tetrahydrofuran in the monoclinic *C*2/*c* space group. The Cl^-^ counter ion is separated by 4.197 Å from the Pt, showing that it is not coordinated. The [Pt(dppp)_2_]^2+^ ions are arranged in two-dimensional layers, with the Cl^-^ anions and solvent between the layers.

### S2. Experimental

[PtCl_2_(SEt_2_)_2_] (50 mg, 0.11 mmol) was dissolved in CDCl_3_ (0.5 ml) in an NMR tube under Ar and 5 equiv. bicyclopropylidene added (50 µ*L*, 0.54 mmol). The reaction was stirred for 5 days, resulting in the formation of *trans*-[Pt(C(CH_2_)_2_C(CH_2_)_2_Cl)Cl(SEt_2_)_2_]. The solution was frozen in liquid N_2_, and a solution of dppp (90 mg, 0.22 mmol) in CDCl_3_ (0.5 ml) added. Crystals of [Pt(dppp)_2_]Cl_2_ formed as the solution warmed to RT.

### S3. Refinement

All H atoms were positioned geometrically and refined using a riding model, with aromatic C—H = 0.93 Å, methylene C—H = 0.97 Å, and tertiary C—H = 0.98 Å. *U*_iso_(H) = 1.2. A chloroform solvent molecule was found to be disordered about a 2-fold axis, and was refined by suppressing the symmetry restriction with a 'PART -1' instruction.

### Crystal data

**Table:**

[Pt(C_27_H_26_P_2_)_2_]Cl_2_·5CHCl_3_	*F*(000) = 3352.00
*M**_r_* = 1687.67	*D*_x_ = 1.664 Mg m^−^^3^
Orthorhombic, *P**c**c**n*	Mo *K*α radiation, λ = 0.71073 Å
Hall symbol: -P 2ab 2ac	Cell parameters from 9982 reflections
*a* = 26.2042 (9) Å	θ = 2.4–30.5°
*b* = 15.3120 (5) Å	µ = 2.89 mm^−^^1^
*c* = 16.7930 (5) Å	*T* = 160 K
*V* = 6738.0 (4) Å^3^	Block, colourless
*Z* = 4	0.6 × 0.38 × 0.28 mm

### Data collection

**Table:**

Bruker APEXII CCD diffractometer	10325 independent reflections
Radiation source: fine-focus sealed tube	7580 reflections with *I* > 2σ(*I*)
Graphite monochromator	*R*_int_ = 0.063
φ and ω scans	θ_max_ = 30.6°, θ_min_ = 2.4°
Absorption correction: multi-scan (*SADABS*; Bruker, 2007)	*h* = −37→37
*T*_min_ = 0.562, *T*_max_ = 0.746	*k* = −21→21
179974 measured reflections	*l* = −23→24

### Refinement

**Table:**

Refinement on *F*^2^	Primary atom site location: structure-invariant direct methods
Least-squares matrix: full	Secondary atom site location: difference Fourier map
*R*[*F*^2^ > 2σ(*F*^2^)] = 0.037	Hydrogen site location: inferred from neighbouring sites
*wR*(*F*^2^) = 0.073	H-atom parameters constrained
*S* = 1.20	*w* = 1/[σ^2^(*F*_o_^2^) + (0.*P*)^2^ + 21.821*P*] where *P* = (*F*_o_^2^ + 2*F*_c_^2^)/3
10325 reflections	(Δ/σ)_max_ = 0.001
385 parameters	Δρ_max_ = 0.73 e Å^−^^3^
0 restraints	Δρ_min_ = −0.97 e Å^−^^3^

### Special details

**Table:**

Geometry. All e.s.d.'s (except the e.s.d. in the dihedral angle between two l.s. planes) are estimated using the full covariance matrix. The cell e.s.d.'s are taken into account individually in the estimation of e.s.d.'s in distances, angles and torsion angles; correlations between e.s.d.'s in cell parameters are only used when they are defined by crystal symmetry. An approximate (isotropic) treatment of cell e.s.d.'s is used for estimating e.s.d.'s involving l.s. planes.
Refinement. Refinement of *F*^2^ against ALL reflections. The weighted *R*-factor *wR* and goodness of fit *S* are based on *F*^2^, conventional *R*-factors *R* are based on *F*, with *F* set to zero for negative *F*^2^. The threshold expression of *F*^2^ > σ(*F*^2^) is used only for calculating *R*-factors(gt) *etc*. and is not relevant to the choice of reflections for refinement. *R*-factors based on *F*^2^ are statistically about twice as large as those based on *F*, and *R*- factors based on ALL data will be even larger.

### Fractional atomic coordinates and isotropic or equivalent isotropic displacement parameters (Å^2^)

**Table:**

	*x*	*y*	*z*	*U*_iso_*/*U*_eq_	Occ. (<1)
Pt1	0.5000	0.0000	0.0000	0.01283 (4)	
P2	0.49876 (3)	0.10087 (5)	0.10661 (4)	0.01623 (14)	
P1	0.48568 (3)	−0.11136 (5)	0.09623 (4)	0.01524 (15)	
C4	0.41837 (12)	−0.1208 (2)	0.11952 (18)	0.0178 (6)	
C3	0.52924 (13)	0.0645 (2)	0.19883 (18)	0.0210 (6)	
H3A	0.5648	0.0519	0.1875	0.025*	
H3B	0.5284	0.1123	0.2367	0.025*	
C18	0.53826 (15)	0.3596 (2)	0.1019 (2)	0.0278 (8)	
H18	0.5219	0.4133	0.1061	0.033*	
C1	0.51746 (13)	−0.0980 (2)	0.19268 (17)	0.0194 (6)	
H1A	0.5082	−0.1472	0.2261	0.023*	
H1B	0.5540	−0.1009	0.1841	0.023*	
C21	0.58744 (13)	0.1990 (2)	0.0910 (2)	0.0234 (7)	
H21	0.6040	0.1453	0.0886	0.028*	
C2	0.50582 (13)	−0.0150 (2)	0.23790 (17)	0.0211 (7)	
H2A	0.5188	−0.0202	0.2918	0.025*	
H2B	0.4691	−0.0074	0.2410	0.025*	
C9	0.40155 (13)	−0.1654 (2)	0.18772 (19)	0.0219 (7)	
H9	0.4251	−0.1879	0.2237	0.026*	
C22	0.43463 (13)	0.1321 (2)	0.13539 (19)	0.0203 (6)	
C14	0.49389 (16)	−0.3787 (2)	0.0796 (2)	0.0299 (8)	
H14	0.4719	−0.4262	0.0833	0.036*	
C5	0.38263 (13)	−0.0870 (2)	0.0673 (2)	0.0239 (7)	
H5	0.3934	−0.0568	0.0222	0.029*	
C8	0.34994 (14)	−0.1758 (3)	0.2012 (2)	0.0294 (8)	
H8	0.3389	−0.2057	0.2461	0.035*	
C16	0.53416 (12)	0.2026 (2)	0.09712 (18)	0.0183 (6)	
C19	0.59096 (16)	0.3563 (3)	0.0944 (2)	0.0316 (8)	
H19	0.6099	0.4077	0.0932	0.038*	
C17	0.50980 (13)	0.2833 (2)	0.10331 (18)	0.0217 (7)	
H17	0.4745	0.2859	0.1084	0.026*	
C11	0.56005 (14)	−0.2376 (2)	0.0705 (2)	0.0251 (7)	
H11	0.5824	−0.1905	0.0679	0.030*	
C10	0.50772 (13)	−0.22317 (19)	0.07887 (17)	0.0189 (6)	
C6	0.33060 (14)	−0.0978 (3)	0.0817 (2)	0.0352 (9)	
H6	0.3068	−0.0752	0.0463	0.042*	
C12	0.57854 (16)	−0.3223 (3)	0.0660 (2)	0.0326 (9)	
H12	0.6134	−0.3320	0.0598	0.039*	
C15	0.47444 (14)	−0.2942 (2)	0.08337 (18)	0.0214 (7)	
H15	0.4395	−0.2852	0.0888	0.026*	
C23	0.39470 (13)	0.1199 (2)	0.0827 (2)	0.0236 (7)	
H23	0.4008	0.0938	0.0336	0.028*	
C20	0.61522 (15)	0.2762 (2)	0.0886 (2)	0.0291 (8)	
H20	0.6505	0.2741	0.0830	0.035*	
C7	0.31462 (15)	−0.1420 (3)	0.1484 (2)	0.0352 (9)	
H7	0.2799	−0.1492	0.1581	0.042*	
C13	0.54565 (17)	−0.3923 (3)	0.0706 (2)	0.0350 (9)	
H13	0.5583	−0.4489	0.0676	0.042*	
C26	0.37622 (16)	0.1975 (3)	0.2287 (3)	0.0365 (9)	
H26	0.3698	0.2235	0.2778	0.044*	
C24	0.34563 (15)	0.1462 (3)	0.1022 (3)	0.0343 (9)	
H24	0.3189	0.1377	0.0665	0.041*	
C27	0.42495 (15)	0.1714 (3)	0.2095 (2)	0.0288 (8)	
H27	0.4515	0.1798	0.2455	0.035*	
C25	0.33674 (16)	0.1853 (3)	0.1753 (3)	0.0414 (10)	
H25	0.3039	0.2034	0.1885	0.050*	
Cl1	0.35882 (4)	0.04388 (8)	−0.11105 (5)	0.0380 (2)	
Cl3B	0.31059 (4)	0.01417 (7)	0.32358 (6)	0.0371 (2)	
Cl2A	0.21666 (4)	0.05178 (8)	0.17673 (6)	0.0434 (3)	
Cl2B	0.24294 (5)	−0.13417 (8)	0.34282 (7)	0.0475 (3)	
Cl3A	0.14625 (5)	−0.09321 (7)	0.16878 (7)	0.0430 (2)	
Cl1A	0.11366 (4)	0.07947 (8)	0.12128 (7)	0.0447 (3)	
Cl1B	0.29044 (4)	−0.05640 (10)	0.48005 (6)	0.0523 (3)	
C1B	0.26446 (14)	−0.0355 (3)	0.3849 (2)	0.0328 (8)	
H1BA	0.2354	0.0042	0.3907	0.039*	
C1A	0.15290 (14)	0.0195 (3)	0.1870 (2)	0.0304 (8)	
H1AA	0.1420	0.0315	0.2417	0.036*	
C1C	0.2657 (3)	0.2740 (5)	0.4313 (4)	0.0282 (15)	0.5
H1C	0.2954	0.3115	0.4218	0.034*	0.5
Cl3C	0.23595 (11)	0.2360 (3)	0.34548 (14)	0.0622 (9)	0.5
Cl1C	0.2171 (5)	0.3222 (11)	0.4886 (6)	0.076 (3)	0.5
Cl2C	0.2802 (3)	0.1693 (9)	0.4893 (3)	0.0494 (17)	0.5

### Atomic displacement parameters (Å^2^)

**Table:**

	*U*^11^	*U*^22^	*U*^33^	*U*^12^	*U*^13^	*U*^23^
Pt1	0.01516 (6)	0.01052 (6)	0.01280 (6)	0.00000 (7)	0.00067 (6)	0.00078 (6)
P2	0.0207 (4)	0.0126 (3)	0.0154 (3)	−0.0020 (3)	0.0032 (3)	−0.0003 (3)
P1	0.0192 (4)	0.0124 (3)	0.0141 (3)	0.0011 (3)	0.0022 (3)	0.0021 (3)
C4	0.0193 (15)	0.0149 (15)	0.0190 (14)	−0.0017 (12)	0.0029 (11)	−0.0026 (11)
C3	0.0254 (17)	0.0203 (16)	0.0174 (14)	−0.0028 (13)	−0.0029 (12)	0.0006 (12)
C18	0.044 (2)	0.0149 (16)	0.0248 (17)	−0.0045 (15)	0.0073 (15)	−0.0037 (13)
C1	0.0232 (15)	0.0188 (15)	0.0163 (13)	0.0000 (13)	−0.0023 (11)	0.0029 (11)
C21	0.0261 (17)	0.0215 (17)	0.0227 (15)	−0.0029 (14)	−0.0008 (13)	0.0006 (13)
C2	0.0278 (18)	0.0213 (17)	0.0141 (12)	−0.0034 (13)	−0.0035 (12)	0.0021 (10)
C9	0.0237 (17)	0.0212 (16)	0.0208 (15)	0.0015 (13)	0.0028 (12)	0.0020 (12)
C22	0.0258 (17)	0.0141 (15)	0.0211 (15)	−0.0009 (13)	0.0056 (12)	0.0026 (12)
C14	0.049 (2)	0.0157 (15)	0.0253 (16)	−0.0002 (16)	0.0086 (16)	0.0042 (12)
C5	0.0250 (17)	0.0251 (18)	0.0216 (15)	−0.0006 (14)	−0.0015 (13)	0.0039 (13)
C8	0.0276 (19)	0.033 (2)	0.0280 (17)	−0.0057 (16)	0.0099 (14)	−0.0017 (15)
C16	0.0240 (16)	0.0164 (15)	0.0146 (13)	−0.0049 (12)	0.0009 (11)	−0.0003 (11)
C19	0.043 (2)	0.0238 (18)	0.0276 (17)	−0.0149 (17)	0.0017 (16)	−0.0028 (15)
C17	0.0293 (19)	0.0170 (15)	0.0188 (14)	0.0002 (13)	0.0070 (12)	0.0005 (11)
C11	0.0303 (19)	0.0194 (17)	0.0255 (16)	0.0025 (14)	0.0090 (14)	0.0037 (13)
C10	0.0283 (19)	0.0106 (13)	0.0179 (13)	0.0016 (12)	0.0056 (12)	0.0030 (10)
C6	0.0229 (18)	0.046 (2)	0.036 (2)	0.0016 (17)	−0.0060 (15)	0.0019 (18)
C12	0.034 (2)	0.0257 (19)	0.038 (2)	0.0122 (16)	0.0134 (16)	0.0076 (16)
C15	0.0292 (18)	0.0194 (16)	0.0156 (14)	0.0002 (13)	0.0032 (12)	0.0016 (12)
C23	0.0254 (17)	0.0175 (16)	0.0280 (17)	0.0003 (13)	0.0041 (13)	0.0015 (13)
C20	0.0284 (19)	0.0282 (19)	0.0307 (18)	−0.0084 (15)	−0.0007 (15)	0.0007 (15)
C7	0.0206 (18)	0.043 (2)	0.042 (2)	−0.0021 (17)	0.0045 (16)	−0.0016 (18)
C13	0.053 (3)	0.0189 (18)	0.0335 (19)	0.0140 (18)	0.0129 (17)	0.0048 (15)
C26	0.037 (2)	0.033 (2)	0.039 (2)	0.0013 (18)	0.0192 (18)	−0.0097 (17)
C24	0.0241 (19)	0.031 (2)	0.047 (2)	−0.0007 (16)	0.0024 (17)	0.0015 (18)
C27	0.031 (2)	0.031 (2)	0.0245 (16)	−0.0018 (16)	0.0088 (14)	−0.0058 (14)
C25	0.027 (2)	0.035 (2)	0.063 (3)	0.0041 (17)	0.018 (2)	−0.001 (2)
Cl1	0.0287 (5)	0.0622 (7)	0.0231 (4)	−0.0202 (5)	−0.0050 (3)	0.0069 (4)
Cl3B	0.0336 (5)	0.0431 (6)	0.0346 (5)	0.0032 (4)	0.0003 (4)	0.0038 (4)
Cl2A	0.0321 (5)	0.0565 (7)	0.0417 (5)	−0.0104 (5)	−0.0056 (4)	0.0056 (5)
Cl2B	0.0446 (6)	0.0378 (6)	0.0602 (7)	−0.0017 (5)	0.0086 (5)	−0.0088 (5)
Cl3A	0.0497 (6)	0.0355 (6)	0.0437 (5)	−0.0053 (5)	−0.0016 (5)	0.0020 (4)
Cl1A	0.0346 (5)	0.0484 (7)	0.0510 (6)	0.0033 (5)	−0.0021 (5)	0.0146 (5)
Cl1B	0.0340 (5)	0.0915 (10)	0.0314 (5)	0.0023 (6)	0.0049 (4)	0.0052 (5)
C1B	0.0246 (19)	0.038 (2)	0.0358 (19)	0.0078 (16)	0.0004 (15)	−0.0033 (17)
C1A	0.031 (2)	0.038 (2)	0.0219 (16)	−0.0022 (16)	0.0035 (14)	0.0008 (14)
C1C	0.024 (4)	0.030 (4)	0.030 (3)	0.000 (3)	−0.004 (3)	−0.001 (3)
Cl3C	0.071 (3)	0.073 (3)	0.0421 (11)	0.0157 (19)	−0.0230 (11)	−0.0151 (14)
Cl1C	0.079 (6)	0.076 (5)	0.074 (5)	0.046 (4)	0.006 (4)	−0.009 (3)
Cl2C	0.045 (3)	0.076 (4)	0.027 (2)	0.041 (3)	0.0020 (18)	0.005 (2)

### Geometric parameters (Å, º)

**Table:**

Pt1—P2	2.3648 (7)	C5—C6	1.395 (5)
Pt1—P2^i^	2.3648 (7)	C8—C7	1.381 (6)
Pt1—P1	2.3790 (8)	C16—C17	1.395 (4)
Pt1—P1^i^	2.3790 (8)	C19—C20	1.385 (5)
P2—C3	1.829 (3)	C11—C10	1.396 (5)
P2—C22	1.813 (3)	C11—C12	1.387 (5)
P2—C16	1.820 (3)	C10—C15	1.396 (4)
P1—C4	1.812 (3)	C6—C7	1.374 (6)
P1—C1	1.833 (3)	C12—C13	1.377 (6)
P1—C10	1.830 (3)	C23—C24	1.386 (5)
C4—C9	1.404 (4)	C26—C27	1.376 (5)
C4—C5	1.384 (5)	C26—C25	1.382 (6)
C3—C2	1.513 (4)	C24—C25	1.386 (6)
C18—C19	1.388 (5)	Cl3B—C1B	1.761 (4)
C18—C17	1.387 (5)	Cl2A—C1A	1.751 (4)
C1—C2	1.512 (4)	Cl2B—C1B	1.760 (4)
C21—C16	1.401 (5)	Cl3A—C1A	1.761 (4)
C21—C20	1.390 (5)	Cl1A—C1A	1.766 (4)
C9—C8	1.380 (5)	Cl1B—C1B	1.765 (4)
C22—C23	1.382 (5)	C1C—Cl3C	1.739 (7)
C22—C27	1.405 (5)	C1C—Cl1C	1.759 (13)
C14—C15	1.391 (5)	C1C—Cl2C	1.914 (14)
C14—C13	1.381 (6)		
			
P2—Pt1—P2^i^	180.0	C4—C5—C6	120.5 (3)
P2^i^—Pt1—P1	92.77 (3)	C9—C8—C7	120.6 (3)
P2—Pt1—P1	87.23 (3)	C21—C16—P2	118.7 (3)
P2—Pt1—P1^i^	92.77 (3)	C17—C16—P2	121.2 (2)
P2^i^—Pt1—P1^i^	87.23 (3)	C17—C16—C21	119.8 (3)
P1^i^—Pt1—P1	180.0	C20—C19—C18	119.7 (3)
C3—P2—Pt1	115.85 (11)	C18—C17—C16	119.9 (3)
C22—P2—Pt1	112.76 (11)	C12—C11—C10	119.8 (3)
C22—P2—C3	105.04 (15)	C11—C10—P1	118.3 (2)
C22—P2—C16	105.67 (15)	C11—C10—C15	119.7 (3)
C16—P2—Pt1	119.06 (10)	C15—C10—P1	121.5 (3)
C16—P2—C3	96.45 (15)	C7—C6—C5	119.9 (4)
C4—P1—Pt1	110.94 (11)	C13—C12—C11	120.4 (4)
C4—P1—C1	105.09 (15)	C14—C15—C10	119.5 (3)
C4—P1—C10	105.47 (15)	C22—C23—C24	120.8 (3)
C1—P1—Pt1	116.67 (11)	C19—C20—C21	120.8 (3)
C10—P1—Pt1	120.83 (10)	C6—C7—C8	120.2 (4)
C10—P1—C1	95.82 (15)	C12—C13—C14	120.2 (3)
C9—C4—P1	121.3 (3)	C27—C26—C25	120.2 (4)
C5—C4—P1	119.5 (2)	C25—C24—C23	119.3 (4)
C5—C4—C9	119.1 (3)	C26—C27—C22	120.0 (4)
C2—C3—P2	115.8 (2)	C26—C25—C24	120.5 (4)
C17—C18—C19	120.4 (3)	Cl3B—C1B—Cl1B	110.0 (2)
C2—C1—P1	116.5 (2)	Cl2B—C1B—Cl3B	110.8 (2)
C20—C21—C16	119.4 (3)	Cl2B—C1B—Cl1B	109.4 (2)
C1—C2—C3	112.1 (3)	Cl2A—C1A—Cl3A	110.8 (2)
C8—C9—C4	119.8 (3)	Cl2A—C1A—Cl1A	110.3 (2)
C23—C22—P2	119.7 (2)	Cl3A—C1A—Cl1A	110.1 (2)
C23—C22—C27	119.2 (3)	Cl3C—C1C—Cl1C	105.6 (5)
C27—C22—P2	121.1 (3)	Cl3C—C1C—Cl2C	103.3 (5)
C13—C14—C15	120.3 (3)	Cl1C—C1C—Cl2C	102.5 (6)
			
Pt1—P2—C3—C2	−61.9 (3)	C21—C16—C17—C18	1.2 (5)
Pt1—P2—C22—C23	−19.8 (3)	C9—C4—C5—C6	−0.7 (5)
Pt1—P2—C22—C27	162.4 (3)	C9—C8—C7—C6	0.2 (6)
Pt1—P2—C16—C21	−64.6 (3)	C22—P2—C3—C2	63.2 (3)
Pt1—P2—C16—C17	121.4 (2)	C22—P2—C16—C21	167.4 (3)
Pt1—P1—C4—C9	−163.8 (2)	C22—P2—C16—C17	−6.6 (3)
Pt1—P1—C4—C5	19.2 (3)	C22—C23—C24—C25	0.2 (6)
Pt1—P1—C1—C2	57.5 (3)	C5—C4—C9—C8	0.8 (5)
Pt1—P1—C10—C11	62.2 (3)	C5—C6—C7—C8	−0.1 (6)
Pt1—P1—C10—C15	−125.6 (2)	C16—P2—C3—C2	171.3 (3)
P2—C3—C2—C1	72.9 (3)	C16—P2—C22—C23	111.8 (3)
P2—C22—C23—C24	−177.6 (3)	C16—P2—C22—C27	−65.9 (3)
P2—C22—C27—C26	177.6 (3)	C16—C21—C20—C19	1.8 (5)
P2—C16—C17—C18	175.1 (2)	C19—C18—C17—C16	0.0 (5)
P1—C4—C9—C8	−176.2 (3)	C17—C18—C19—C20	−0.3 (5)
P1—C4—C5—C6	176.4 (3)	C11—C10—C15—C14	0.2 (5)
P1—C1—C2—C3	−70.5 (3)	C11—C12—C13—C14	0.1 (6)
P1—C10—C15—C14	−172.0 (2)	C10—P1—C4—C9	63.7 (3)
C4—P1—C1—C2	−65.8 (3)	C10—P1—C4—C5	−113.3 (3)
C4—P1—C10—C11	−171.2 (2)	C10—P1—C1—C2	−173.6 (3)
C4—P1—C10—C15	1.1 (3)	C10—C11—C12—C13	−0.7 (6)
C4—C9—C8—C7	−0.6 (6)	C12—C11—C10—P1	173.0 (3)
C4—C5—C6—C7	0.3 (6)	C12—C11—C10—C15	0.6 (5)
C3—P2—C22—C23	−146.9 (3)	C15—C14—C13—C12	0.7 (6)
C3—P2—C22—C27	35.4 (3)	C23—C22—C27—C26	−0.2 (5)
C3—P2—C16—C21	59.8 (3)	C23—C24—C25—C26	−0.5 (6)
C3—P2—C16—C17	−114.2 (3)	C20—C21—C16—P2	−176.1 (3)
C18—C19—C20—C21	−0.6 (6)	C20—C21—C16—C17	−2.1 (5)
C1—P1—C4—C9	−36.9 (3)	C13—C14—C15—C10	−0.8 (5)
C1—P1—C4—C5	146.2 (3)	C27—C22—C23—C24	0.2 (5)
C1—P1—C10—C11	−63.7 (3)	C27—C26—C25—C24	0.4 (7)
C1—P1—C10—C15	108.5 (3)	C25—C26—C27—C22	−0.1 (6)

Symmetry code: (i) −*x*+1, −*y*, −*z*.
