# Supplementary figures and images for: Crystal structure of bis­[1,3-bis­(di­phenyl­phosphan­yl)propane-κ2 P,P′]platinum(II) dichloride chloro­form penta­solvate
Source: Acta Crystallogr E Crystallogr Commun. 2015 Jan 28;71(Pt 2):m37. doi: 10.1107/S205698901500136X (PMC4384563; doi:10.1107/S205698901500136X)

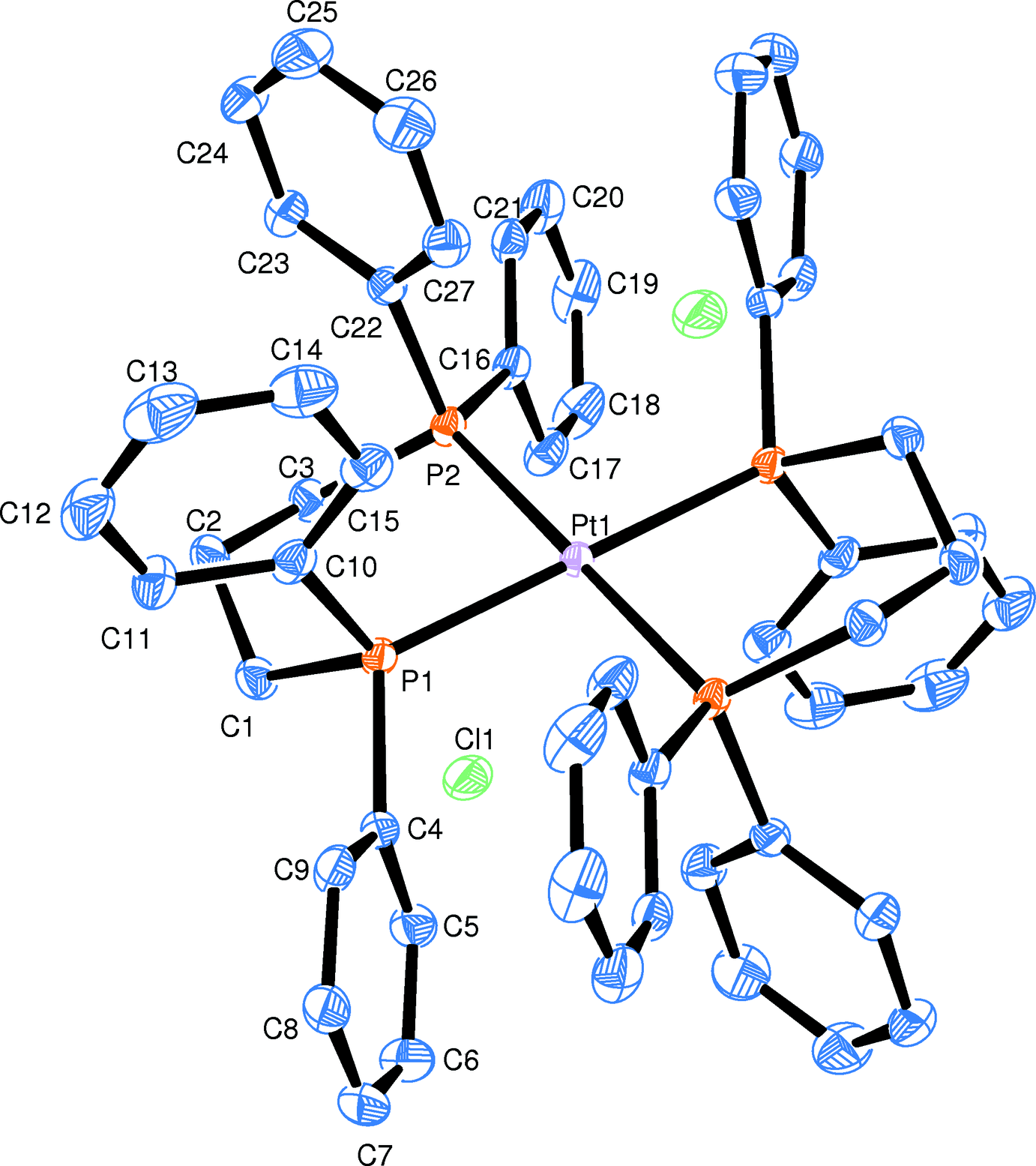

Supplement: Supplementary file 3 [file e-71-00m37-fig1.tif]
